# Analysis and prediction of single-stranded and double-stranded DNA binding proteins based on protein sequences

**DOI:** 10.1186/s12859-017-1715-8

**Published:** 2017-06-12

**Authors:** Wei Wang, Lin Sun, Shiguang Zhang, Hongjun Zhang, Jinling Shi, Tianhe Xu, Keliang Li

**Affiliations:** 1 College of Computer and Information Engineering, Henan Normal University, Xinxiang, Henan Province 453007 China; 2 Laboratory of Computation Intelligence and Information Processing, Engineering Technology Research Center for Computing Intelligence and Data Mining, Xinxiang, Henan Province 453007 China; 3 School of Aviation Engineering, Anyang University, Anyang, Henan Province 455000 China; 4 School of International Education, Xuchang University, Xuchang, Henan Province 461000 China

**Keywords:** SSBs (Single-stranded DNA-binding proteins), DSBs (Double-stranded DNA-binding proteins), Binding specificity, Protein sequence

## Abstract

**Background:**

DNA-binding proteins perform important functions in a great number of biological activities. DNA-binding proteins can interact with ssDNA (single-stranded DNA) or dsDNA (double-stranded DNA), and DNA-binding proteins can be categorized as single-stranded DNA-binding proteins (SSBs) and double-stranded DNA-binding proteins (DSBs). The identification of DNA-binding proteins from amino acid sequences can help to annotate protein functions and understand the binding specificity.

In this study, we systematically consider a variety of schemes to represent protein sequences: OAAC (overall amino acid composition) features, dipeptide compositions, PSSM (position-specific scoring matrix profiles) and split amino acid composition (SAA), and then we adopt SVM (support vector machine) and RF (random forest) classification model to distinguish SSBs from DSBs.

**Results:**

Our results suggest that some sequence features can significantly differentiate DSBs and SSBs. Evaluated by 10 fold cross-validation on the benchmark datasets, our prediction method can achieve the accuracy of 88.7% and AUC (area under the curve) of 0.919. Moreover, our method has good performance in independent testing.

**Conclusions:**

Using various sequence-derived features, a novel method is proposed to distinguish DSBs and SSBs accurately. The method also explores novel features, which could be helpful to discover the binding specificity of DNA-binding proteins.

**Electronic supplementary material:**

The online version of this article (doi:10.1186/s12859-017-1715-8) contains supplementary material, which is available to authorized users.

## Background

Proteins-DNA interaction is important for a great number of biological processes such as DNA replication, transcription, DNA repair and gene expression [1–4], etc. DNA-binding proteins contain essential protein-DNA binding domains, and they have specific or general affinities for either ssDNA or dsDNA [5–7]. Currently, X-ray crystallography, NMR and filter binding assays have been used to dissect structural features [8–10], multiple domain structures of SSBs [11], uncover the biological functions [12–15], etc. However, wet methods of identifying DSBs and SSBs are relatively expensive and time-consuming. Therefore, a reliable and effective computational method is an urgent task, and computational method plays a crucial role in protein function annotation and the identification of proteins. However, a great number of computational methods have been focused on analyzing the specific binding sites of DSBs [16–22], classification of DNA binding proteins [23–28] and protein-DNA binding specificities [29] etc. But few methods pay attention to the large-scale identification of DSBs and SSBs. In our previous work [30], we constructed a SVM prediction model to classify DSBs and SSBs based on the structure information. Although structure-based methods can produce high-accuracy performances, they can’t be applied in high-throughput function annotation because limited structures are known. In contrast, the prediction based on sequence information has more potential use in practice. In this work, we predict whether a protein binds ssDNA or dsDNA without relying on the geometry of the protein. The protein sequence can provide lots of information for predicting protein function [31]. At present, the most familiar methods for predicting protein function involve sequence features [32]. Many methods are employed to predict protein function classes, such as homology detection, sequence patterns, structural similarity, and so on. However, few computational works have studied the sequence features and identify SSBs and DSBs sequences. The recent study [8] shows that SSBs bind with specifically and non-specifically to ssDNA and SSBs have lower sequence conservation. Some DSBs with similar functions have common subsequences, and diverse DSBs involved in different functions seem to have lower conserved subsequences [33]. Recognizing DNA-binding protein sequences helps to realize the implications of properties of proteins and reveal the undiscovered protein features, which help to understand the mechanism of protein-DNA interactions. [34–36].

**Fig. 1:**
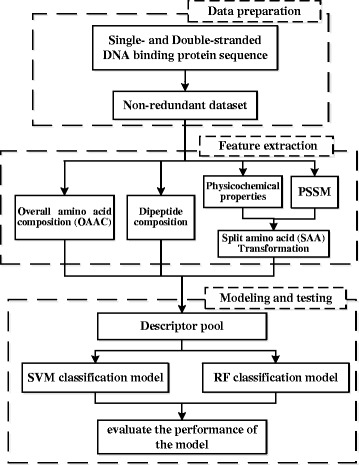
The whole workflow of our method

Here, we propose a novel method to predict DSBs or SSBs by using the SVM algorithm and random forest (RF) algorithm with various sequence-derived features. Specifically, consider a variety of sequence-derived features, including OAAC, PSSM, dipeptide composition, and physicochemical properties, which can provide diverse information to differentiate ssDNAs from dsDNAs. Fig. 1 shows the workflow of our method. In the computational experiments, our model achieves MCC of 0.647 (Matthew’s correlation coefficient), accuracy of 0.887, sensitivity of 0.908 and specificity of 0.788 based on 10-fold cross-validation, respectively. The results show that our method can perform well in predicting SSBs or DSBs for novel proteins.

## Methods

### Training datasets

In this study, DNA-binding proteins were obtained from UniProtKB/Swiss-Prot (www.uniprot.org). The dataset consists of 2136 DSBs and 339 SSBs which are extracted from literature and manually reviewed entries (Additional file 1). Then we used the CD_HIT toolkit [37] to extract sequences with non-redundant proteins (Sequence identity cut-off 0.7). Finally, we obtained 873 DSBs and 183 SSBs (Additional file 2), which is called Uniprot1065. To deal with the unbalanced datasets, a larger number of samples were selected by down-sampling methods during the training process. We obtained a “Negative sample” dataset by randomly selecting subsequences which has the equal size of the SSBs dataset from DSBs dataset.

### Independent datasets

Further, an independent dataset was obtained from PDB (www.rcsb.org/pdb/) to evaluate the performance in predicting novel proteins. PISCES is used (http://dunbrack.fccc.edu/Guoli/PISCES.php) to obtain the non-redundant PDB401 dataset, in which every structure is determined by X-ray or NMR, and resolution better than 3 Å. The sequence similarity is lower than 30%, and the sequence length is higher than 40 residues. In addition, we checked the similarity between the training and independent test sets. We also used the CD_HIT toolkit to extract the non-redundant proteins in the independent dataset. As a result, we obtained the non-redundant independent set of 125 DSBs and 41 SSBs (Additional file 3).

### Protein features

Sequence-derived features can reflect the characteristics of the protein sequences. Here, we consider four types of sequence-derived features, including overall amino acid composition (OAAC), dipeptide composition, PSSM profiles and physicochemical properties. The overall amino acid composition expresses the global descriptors of proteins. Dipeptide composition is the detailed descriptors of sequences and the other two kinds of properties are transformed with the split amino acid composition for describing local features of sequence. The details of features are described as follows.

### Overall amino acid composition (OAAC)’

The OAAC method is a 20-dimensional descriptor of a protein sequence, which describes the frequencies of amino acids in the sequence. It is defined as the follow:

1 $$ {p}_i=\frac{n_i}{L}\kern1em \left( i=1,2\cdots, 20\right) $$

Where *p*_*i*_ is the occurrence frequency of the *i-th* amino acids occurrence, *L* is the total sequence length, and *n*_*i*_ is the sum of the *i-th* amino acids in the sequence.

Researches have shown that a better result can be reached by computing the square root of *p*_*i*_ [38]. Therefore, *f*_*i*_ is used for the OAAC features.

2 $$ {f}_i=\sqrt{P_i}\kern1em \left( i=1,2\cdots, 20\right) $$

### Dipeptide composition

**Fig. 2:**
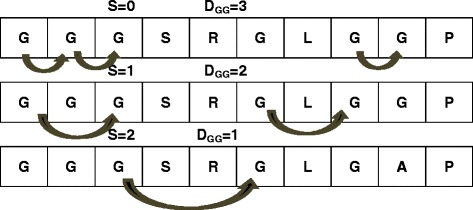
Schematic representation for three kinds of dipeptide composition. The dipeptide compositions are calculated for every two residues in case of 0, 1 and 2 of intervals respectively

Dipeptide component is an important representation of a protein sequence, and has been widely used in the secondary structure prediction [39], subcellular localization and fold recognition [24]. Dipeptide composition contains two consecutive residues information of each sequence, which has 400 patterns [40]. In this work, three types of dipeptide compositions were calculated for every two residues in case of 0, 1 and 2 of intervals respectively, as illustrated in Fig. 2. The dipeptide composition is defined as:

3 $$ {f}_s\left( i, j\right)=\frac{D_s\left( i, j\right)}{N-1}\kern0.75em \left( i, j=1,2,3\cdots, 20\kern0.75em  s=0,1,2\right) $$

Where *D*_*s*_(*i*, *j*) represents the total of each type of *i* and *j* dipeptides with *s* of intervals where *s* = 0, 1, 2, and *N* is the sequence length of protein. *f*_*s*_(*i*, *j*) is the occurrence frequency of every dipeptides. Finally, we got a total of 1200 dimensional vectors with dipeptides of varying intervals together.

### Physicochemical properties

Physicochemical properties play a major role in analyzing DNA-binding mechanism. AAindex is widely used in many studies of physicochemical properties of amino acids. A great number of algorithms for predicting protein functions had been developed by using physicochemical properties from AAindex. Here, we used 28 AAindex properties (Table 1) which are selected by the Auto-IDPCPs methods [41]. Each protein is represented by a set of 28*L matrix array along with the *L*-residue number.

**Table 1:** The list of AAIndex physicochemical properties we used

ID	AAIndex	ID	AAIndex	ID	AAIndex	ID	AAIndex
39	CHOP780202	102	GEIM800106	229	PALJ810107	401	ZIMJ680104
56	CIDH920103	139	KANM800102	280	QIAN880123	422	AURR980120
58	CIDH920105	146	KLEP840101	299	RACS770103	431	MUNV940103
86	FAUJ880109	147	KRIW710101	321	RADA880108	449	NADH010104
88	FAUJ880111	167	LIFS790101	356	ROSM880102	451	NADH010106
95	FINA910104	178	MEEJ800101	365	SWER830101	512	GUYH850105
100	GEIM800104	214	OOBM770102	399	ZIMJ680102	528	MIYS990104

### PSSM profiles

The PSSM is an important tool to predict protein function, and the PSSM profiles represent the evolution information, which has been widely used in protein function prediction [42]. Here, PSSM profiles are obtained by using PSI-BLAST [43]. The PSSM was calculated by three iterations of PSI-BLAST to search the non-redundant NCBI database based on the substitution matrix of BLOSUM62. The parameter of e-value was set to 0.001. This PSSM scoring matrix has *L* rows and 20 columns, and *L* rows are the sequence length of a protein, and 20 columns represent the occurrence of each kind of 20 amino acids.

### Split amino acid (SAA) transformation

**Fig. 3:**
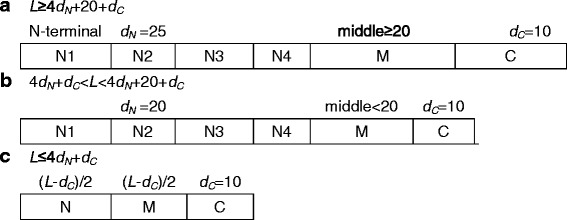
The SAA method defining the N-terminal, middle, and C-terminal regions based on the sequence length *L*. If *L* is no less than 4*d*_*N*_ + 20 + *d*_*c*_, the N-terminal of each part *d*_*N*_ is represented 25 residues in the start region of sequence. The middle regions have more than 20 residues, and the length of the C-terminal regions *d*_*c*_ is set as 10 residues (**a**). If *L* is between 4*d*_*N*_ + *d*_*c*_ and 4*d*_*N*_ + 20 + *d*_*c*_, the N-terminal of each part *d*_*N*_ is represented by 20 residues in the start part. Thus, the middle part has less than 20 residues (**b**). For short sequence, if *L* is no less than 4*d*_*N*_ + *d*_*c*_, the N-terminal part is not divided and the lengths of the middle and N-terminal regions are set as (*L*-*d*_*c*_)/2 (**c**)

SAA transformation was used to describe the local composition of protein sequences [44]. SAA transformation partitions each sequence into three regions: the parts of the N-terminal, middle and C-terminal. The composition of each region is shown in Fig. 3. The variable length sequences were partitioned with a fixed length pattern of the 6 dimensional vectors. The sequences are defined as N-terminal regions, middle regions and C-terminal regions based on their position. For the sequences with varied lengths, we used three definitions to represent the local composition (Fig. 3).

### Classification model and evaluation method

The classification models are built by using SVM and random forest with above mentioned features. SVM models are implemented by the SVM package in Matlab 2012a. The default parameters of SVM are adopted in the experiments. The random forest models are implemented by using Andy Liaw’s Matlab package. The number of trees is set to 3000. Two classifiers are used to build prediction models and then compared. The performances of classification models were evaluated by AUC (area under the ROC curve), F1 (F-measure), Acc (accuracy), Spe (specificity), Sen (sensitivity) and MCC (Matthew’s correlation coefficient).

The 10-fold cross-validation is usually adopted to evaluation performances of classification models. In the 10-fold cross-validation, data is randomly divided into ten equal parts. In each fold, one part is kept for the testing dataset and nine parts are used as the training dataset. In each training dataset, classification models were constructed based on different features and predictions for testing set is given by combining outputs of classification models by majority voting strategy. The ensemble learning is a strategy to improve the performances of classification, and has lots if successful applications [45–54]. The majority voting strategy is a popular way of the ensemble learning, and can combine various sequence-derived to predict single-stranded and double-stranded DNA binding proteins.

## Results and discussion

### OAAC results

**Fig. 4:**
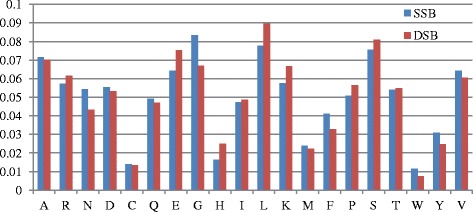
The frequency distributions of 20 kinds of amino acids in DSBs and SSBs

To evaluate the OAAC method, we detected the sequence composition of two kinds of proteins, and the comparisons of the two types of proteins are shown in Fig. 4. DSBs residues have only slightly higher frequency than SSBs, including Arg (R), Lys (K), Glu (E), Pro (P), Ser (S), Leu (L) and His (H). Clearly, the positive charge residues (Arg, His and Lys) in DSBs have a higher level than these of SSBs, and it coincides with the fact that dsDNA strand has higher negative charge than ssDNA strand, and dsDNA has a stabilized double-helix structure while ssDNA presents unwound and irregular helix. Therefore, the positive charges of sequence residues are more enriched to DSBs than SSBs. Asn (N), Gly (G), Phe (F), Tyr (Y) and Val (V) in the SSBs sequences have a higher frequency than those in the DSBs. It is believed that the differences of sequences can be used to distinguish DSBs and SSBs. The OAAC values (20 × *N* dimension matrices, *N* is the number of proteins) of each protein are used as features.

### Dipeptide composition analysis

**Fig. 5:**
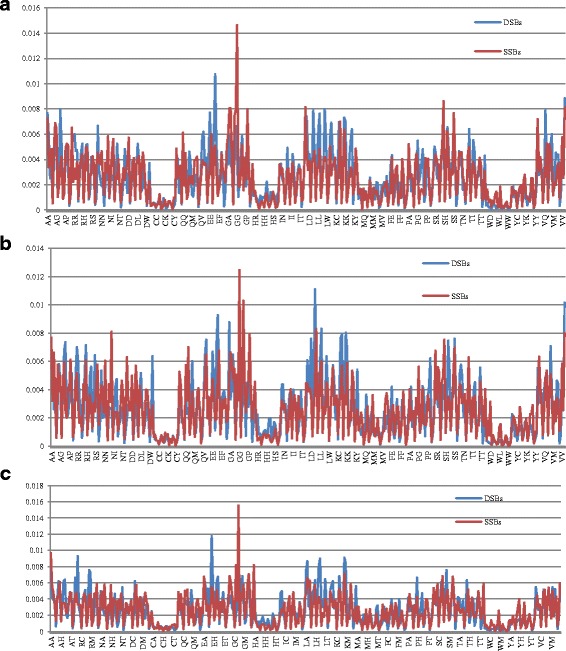
The frequency distributions of paired amino acids in the condition with 0, 1 and 2 of intervals. **a** The dipeptide frequency in case of 0 of intervals. **b** The dipeptide frequency in case of 1 of intervals. **c** The dipeptide frequency in case of 2 of intervals

In the study, dipeptide compositions are used to obtain the global sequence information. The dipeptide compositions is analyzed statistically by computing pairs of amino acids conditions with 0, 1 and 2 of intervals respectively [55]. The dipeptide frequency of 0, 1 and 2 of intervals is shown in Fig. 5. The differences for 16 kinds of dipeptide of frequency are more than 0.003 (AL, RA, EE, EL, GN, GQ, GG, LE, LS, KA, KE, KG, KT, PK, VA and VE) and 2 kinds of dipeptide (ES, EB) are less than 0.005 in Fig. 5a. The frequency differences of 25 kinds of dipeptide are more than 0.003, which are shown in Fig. 5b (AR, AN, AK, RR, RP, RT, NN, NG, NV, DV, QG, EI, EL, EV, GA, GG, GL, LD, LE, KE, KL, PG, PT, SG and SL) and those 6 kinds of dipeptide are less than 0.005 (KL, LE, GG, GL, EI and NG). The frequency difference of 19 kinds of dipeptide are more than 0.003, which are shown in Fig. 5c (RR, RL, NN, NG, DG, DP, QL, EE, EK, GA, GQ, GG, LL, LK, KR, KK, FF, SL and TE) and the 5 kinds of dipeptide are less than 0.005 (GG, GQ, LK, EE and RR). The results show the effectiveness of the dipeptide compositions feature.

The studies on protein-DNA binding have found some related physicochemical properties of amino acid, which were regarded as critical factors of protein-DNA binding mechanism. There are several typical physicochemical properties which are discovered to be associated with protein-DNA binding including charge, hydrophobicity, flexibility, solvent accessibility, polarity, volume and pK, etc. However, it is still unknown that whether those physicochemical properties are associated with the proteins specific binding to dsDNA or ssDNA. It is difficult to screen out related physicochemical properties for predicting SSBs and DSBs specific to biological methods. Therefore, 28 kinds of typical physicochemical properties, which are significantly different in 6 parts of SAA, are selected to analyze DSBs and SSBs. The physicochemical properties were evaluated for revealing protein-DNA interactive mechanism by computational methods. Here, we present a feature analytical method of the physicochemical features. The method is defined as follows.

4 $$ X=\frac{\left|\overline{K_1}-\overline{K_2}\right|}{\mathit{\operatorname{Max}}\left(\overline{K_1},\overline{K_2}\right)} $$

Where *X* is the physicochemical properties difference in rate for DSBs and SSBs. $\overline{K_1}$ and $\overline{K_2}$ are the average for all physicochemical properties in every part of SAA.

**Fig. 6:**
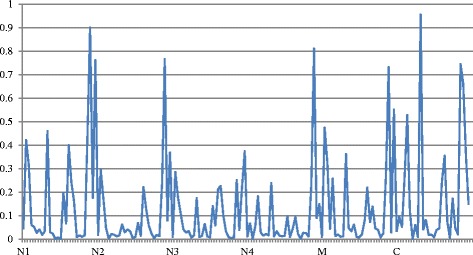
The physicochemical properties different rate in 6 parts of SAA between DSBs and SSBs. SAA transformation partitions each sequence into 6 parts: the parts of the N-terminal (N1, N2, N3 and N4), middle (M) and C-terminal (C)

The differences of physicochemical properties are shown in Fig. 6. According to statistical results, DSBs and SSBs show significant difference in some properties, and the AAindex IDs of physicochemical properties are shown in Table 2. Obviously, five parts share some properties, including RADA880108 (Mean polarity), NADH010104 (Hydropathy scale, 20% accessibility), NADH010106 (Hydropathy scale, 36% accessibility), and four parts share KLEP840101 properties (Net charge), CIDH920103 (Standardized hydrophobicity measures), MIYS990104 (Self-consistent estimation of inter-residue protein) [56]. The N-terminal (N1 and N3) and C-terminal share GUYH850105 (Amino acid side-chain partition energies). There are significant differences with polarity, hydropathy, net charge and protein contact energies between DSBs and SSBs. Therefore, these properties are playing critical roles in selecting the specific binding of dsDNA and ssDNA.

**Table 2:** AAindex IDs of significant differences in different SAA parts

SAAC parts	AAindex IDs of significant differences
N-terminal(N1)	CIDH920103,CIDH920105,KLEP840101,RADA880108,ROSM880102,NADH010106,GUYH850105,MIYS990104
N-terminal(N2)	CIDH920103,RADA880108,NADH010104,NADH010106,MIYS990104
N-terminal(N3)	CIDH920103,RADA880108,ROSM880102,NADH010104,GUYH850105,MIYS990104
N-terminal (N4)	KLEP840101,NADH010104,NADH010106,
Middle(M)	CIDH920103,CIDH920105,FAUJ880111,KLEP840101,RADA880108,NADH010104,NADH010106,MIYS990104
C-terminal(C)	FAUJ880109,FAUJ880111,KLEP840101,RADA880108,ROSM880102,NADH010104,NADH010106,GUYH850105

### Prediction performance of the classifiers

**Fig. 7:**
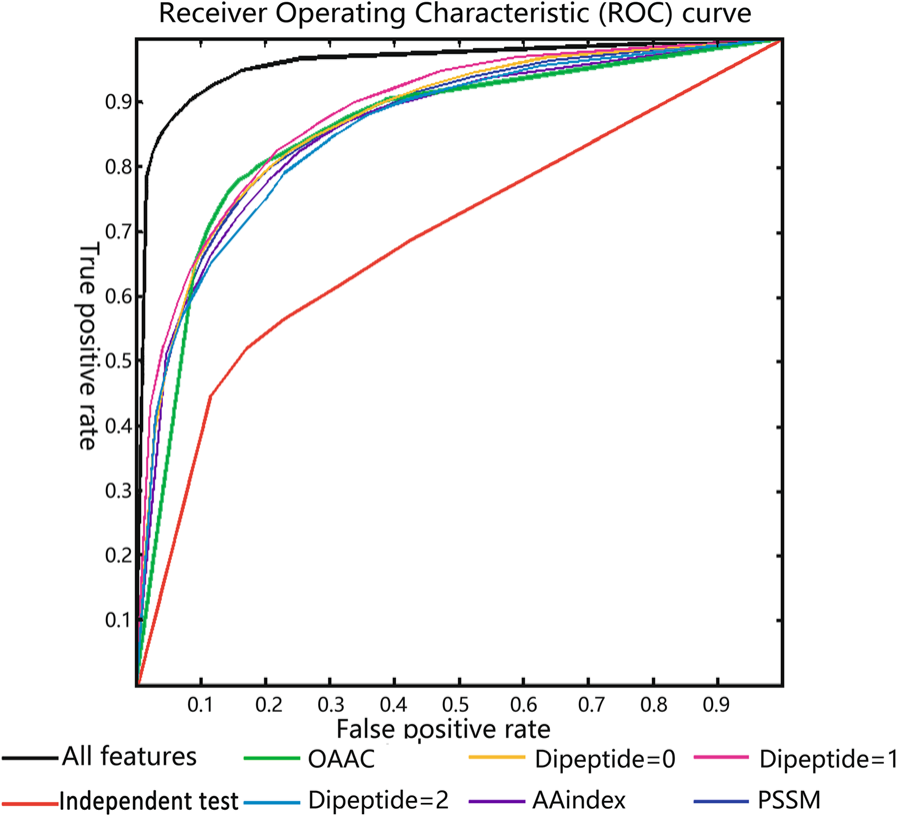
The ROC curve of the SVM model. The ROC plots the summarizing results of the SVM testing in the dataset using the different features. The red curve represents the result of independent test on PDB dataset, and others represent the results of 10-fold cross validation on Uniprot1065 set

**Fig. 8:**
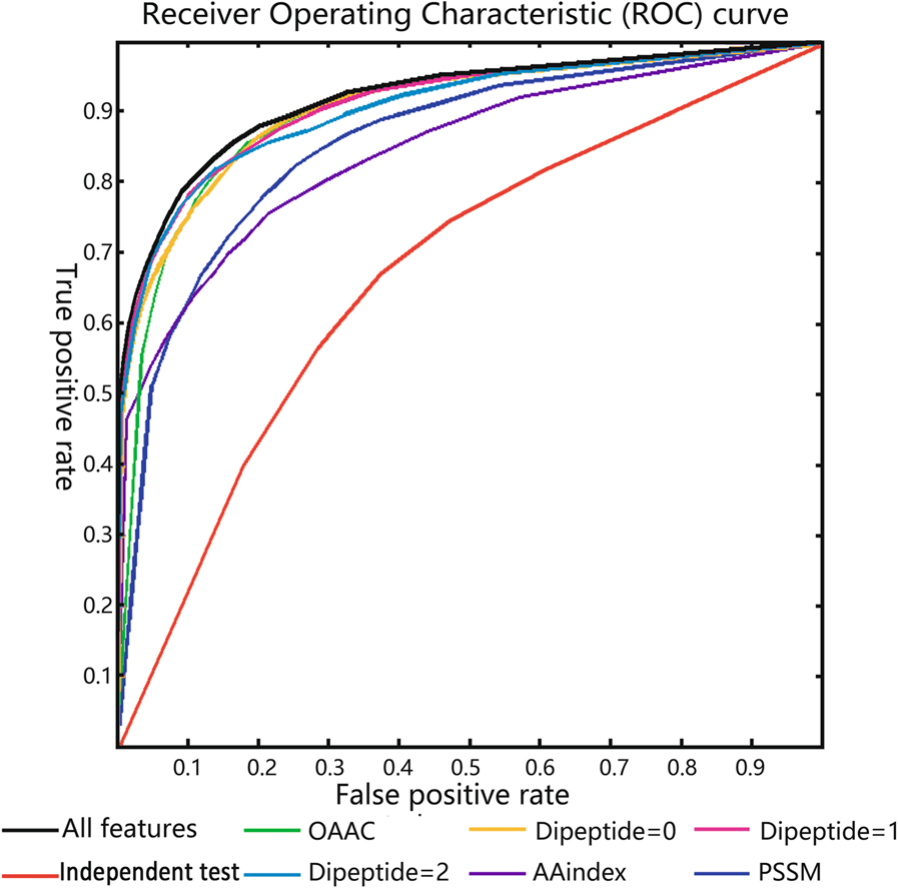
The ROC curve of random forest models. The ROC plots the summarizing results of random forest testing in the dataset using the different features. The red curve represents the result of independent test on PDB dataset, and others represent the results of 10-fold cross validation on Uniprot1065 set

In order to identify whether the selected features can be employed to classify DSBs and SSBs, we constructed the SVM and random forest classifier with different features by 10-fold cross validation and leave-one-out cross validation in Uniprot1065 set. Here, we constructed six predicting models with individual features. ROC plots the summarizing results of the SVM and random forest testing in the dataset using the different features described in Figs. 7 and 8, and the ROC shows that all features dramatically improved the predicted performance. In addition, the results of leave-one-out cross validation are shown in Additional file 4. The Gini importance of each feature type is an importance characteristic parameter in random forest. We tested the Gini importance of each feature, and obtained the average value of Gini values in 10-fold cross-validation, and found out some significant difference in the features. The figures of Gini importance are provided in Additional file 5. For example, we can observe that Leu (L), Gly (G), Phe (F), Asn (N) and Trp (W) are hydrophobic, and have comparatively high Gini importance in Additional file 5: Figure S1. The results show that hydrophobicity may be one of the most significant characteristics between DSBs and SSBs.

From the results of Tables 3 and 4, we can find that all single features can predict SSBs and DSBs with good performance. In experimentations, we found that the best precision is obtained by using multiple features with the highest prediction accuracy of 0.887, SN of 0.908, SP of 0.788 and AUC of 0.919 based on random forest model. The results suggest that the OAAC, PSSM, Dipeptide and AAindex features are important features to predict SSBs from DSBs sequences. Moreover, comparing Table 3 with Table 4, the random forest models also show better classification performance than SVM models. The results show that the random forest may be advantageous to deal with problems with high dimensions and unbalanced samples. Based on the results of individual modules, the predicting models based on *Dipeptide = 1* feature obtained better performance than any other features, which illustrate that the *Dipeptide = 1* feature of the sequences is more crucial to predicting DSBs from SSBs.

**Table 3:** The performance of different kinds of feature descriptors in non-redundant dataset by SVM method

Features	ACC	SN	SP	AUC	MCC	F1
OAAC	0.802	0.801	0.811	0.858	0.505	0.870
Dipeptide = 0	0.797	0.798	0.795	0.870	0.490	0.867
Dipeptide = 1	0.810	0.814	0.791	0.884	0.506	0.877
Dipeptide = 2	0.792	0.801	0.750	0.859	0.459	0.865
AAindex	0.792	0.795	0.779	0.857	0.473	0.864
PSSM	0.795	0.797	0.790	0.863	0.484	0.866
All features	0.860	0.863	0.845	0.923	0.615	0.911

**Table 4:** The performance of different kinds of feature descriptors in non-redundant dataset by random forest method

Features	ACC	SN	SP	AUC	MCC	F1
OAAC	0.849	0.856	0.817	0.900	0.581	0.904
Dipeptide = 0	0.872	0.892	0.780	0.910	0.612	0.921
Dipeptide = 1	0.879	0.900	0.781	0.912	0.625	0.925
Dipeptide = 2	0.870	0.885	0.797	0.908	0.612	0.918
AAindex	0.819	0.844	0.698	0.846	0.475	0.886
PSSM	0.836	0.855	0.744	0.884	0.527	0.896
All features	0.887	0.908	0.788	0.919	0.647	0.930

### Independent test on PDB set

Furthermore, we had done an independent test using PDB401 dataset to validate our method. The results listed in Table 5 demonstrate that SVM classifier obtains a better performance. In SVM model, the combination of all features achieves the good performance with accuracy of 0.642, sensitivity of 0.613, specificity of 0.732, Matthew correlation coefficient of 0.298, and AUC of 0.672, respectively. The random forest method achieves a higher accuracy of 0.727, but the results of random forest methods show some of the biases in sensitivity and specificity. The random forest method achieves a higher sensitivity but a lower specificity, and the SVM model performs better than that of random forest in specificity and AUC. As we know that there is no computational works to identify SSBs and DSBs sequences, therefore we had train a baseline model by permuting the labels of SSBs and DSBs in the training data, and applied this model to predict the independent dataset. The results are shown in Table 5. In addition, we extracted the structures of 727 un-annotated DNA binding proteins from PDB. Then, we used CD-HIT to get the non-redundant set. We finally got 568 un-annotated proteins. The un-annotated proteins are predicted by using the prediction method, and the results are shown in Additional file 6. In general, the result indicates that our method has good generalization abilities in classifying DNA-binding proteins in novel proteins.

**Table 5:** The performance of all feature descriptors with various machine learning algorithms based on independent dataset

Method	Features	ACC	SN	SP	AUC	MCC	F1
Random forest	OAAC	0.697	0.734	0.585	0.660	0.290	0.785
	Dipeptide = 0	0.570	0.557	0.610	0.583	0.144	0.660
	Dipeptide = 1	0.696	0.731	0.590	0.687	0.292	0.784
	Dipeptide = 2	0.546	0.516	0.634	0.575	0.130	0.631
	AAindex	0.703	0.798	0.415	0.607	0.211	0.802
	PSSM	0.703	0.774	0.488	0.631	0.249	0.797
	All features	0.727	0.807	0.488	0.647	0.288	0.816
SVM	All features	0.642	0.613	0.732	0.672	0.298	0.720
Baseline	All features	0.509	0.492	0.558	0.526	0.044	0.591

## Conclusions

In this study, we compile a non-redundant sequence dataset consisting of 873 DSBs and 183 SSBs, and build four kinds of typical features underlying DNA binding proteins sequences. Using the features, we developed SVM-based model and RF-based model to predict SSBs from DSBs sequences. The results confirmed the distinguishing abilities of the features. Interestingly, OAAC, dipeptide compositions and physicochemical properties presents remarkable difference between DSBs and SSBs. The independent test confirms the effectiveness of the model. Based on the sequence-derived features, RF model has a prediction accuracy of 88.7% and AUC of 0.919, and SVM performs better in independent data set. In general, our results indicate that the method can effectively predict DSBs and SSBs sequence to investigate DNA binding protein sequences, and these amino acid properties may be critical to describe the specific binding of a protein for ssDNA or dsDNA molecule.

## Additional files

Additional file 1: This file contains the complete list of UniProt codes for the whole DNA-binding protein sets from UniProtKB/Swiss-Prot (www.uniprot.org). (DOCX 26 kb)

Additional file 2: This file contains the list of UniProt codes for non-redundant DNA-binding protein sets from UniProtKB/Swiss-Prot (www.uniprot.org). (DOCX 19 kb)

Additional file 3: This file contains the list of PDB codes for non-redundant DNA-binding protein independent sets from PDB (www.rcsb.org/pdb/). (DOCX 16 kb)

Additional file 4: This file contains the results of leave-one-out cross validation. (DOCX 24 kb)

Additional file 5: This file contains Gini importance of each feature type in random forest. (XLSX 86 kb)

Additional file 6: This file contains the prediction results for 568 un-annotated proteins. (XLSX 22 kb)

## Abbreviations

Acc: Accuracy
AUC: Area under the curve
DSBs: Double-stranded DNA binding proteins
dsDNA: Double-stranded DNA
F1: F-measure
MCC: Mathews Correlation Coefficient
MCC: Matthew’s correlation coefficient
OAAC: Overall amino acid composition
PSSM: Position-specific scoring matrix
RF: Random forest
ROC: Receiver operating characteristics
SAA: Split amino acid
Sen: Sensitivity
Spe: Specificity
SSBs: Single-stranded DNA binding proteins,
ssDNA: Single-stranded DNA
SVM: Support vector machine
