# Supplementary material for: Analysis and prediction of single-stranded and double-stranded DNA binding proteins based on protein sequences
Source: BMC Bioinformatics. 2017 Jun 12;18:300. doi: 10.1186/s12859-017-1715-8 (PMC5469069; doi:10.1186/s12859-017-1715-8)
Supplement: Supplementary file 1 — This file contains the complete list of UniProt codes for the whole DNA-binding protein sets from UniProtKB/Swiss-Prot (www.uniprot.org). (DOCX 26 kb) [file 12859_2017_1715_MOESM1_ESM.docx]

**Table S1 -** **complete list of UniProt codes for the whole DNA-binding protein sets.**

| **Types** | **ID** |
| --- | --- |
| **DSBs** | P13123, P39476, O75717, Q92466, Q16531, P0A3H0, P08821, P59328, P19436, P61991, O13046, P04498, Q2NB98, P13125, P0ACF8, P21538, P40969, P36659, P0ACF0, P0ACF4, P0A6R3, P32504, P40201, P11938, P35251, Q8TD26, Q63003, Q02486, P08874, P06022, P31441, P29383, P03085, P03084, O22812, Q8L7L5, Q9M2S3, Q9LTA2, Q9SJG4, Q9LZX7, Q8VYJ2, Q8LPN5, A1L4X7, Q940I0, Q6DBQ1, O49662, Q9S7C9, Q9SZ70, O49658, O22130, O82166, Q9SR17, O23620, Q9M9R4, Q8GXB3, Q9SB31, Q9FIR1, Q9FHM5, Q4V3E0, Q8GWQ2, Q9LVB0, Q9C9K7, O80834, P33224, O43918, Q9Z0E3, P60848, P68767, A1A4R9, Q5RJ20, Q6VUC0, P38065, Q2T9K2, Q9N0N3, Q6P0E7, Q91ZK0, Q6VUP9, Q7Z6R9, Q92481, P05549, Q61312, P58197, Q76HI7, Q92754, Q61313, P34056, Q06481, Q06335, Q7YRZ2, P61797, P61799, P61802, P61798, Q7TQC5, Q8MSG8, Q7Z2E3, Q8K4H4, Q7YRZ1, Q7T287, Q9BGQ0, P61800, Q08702, P61801, Q62431, Q3SWY1, Q3U108, Q8BM75, A7ZKT2, Q0TIL5, Q8FI39, P75993, A1AA91, B1XA53, B1IUC4, A7ZZ97, B1LI03, P32447, P34233, Q9LJG8, Q9SYG2, P16951, Q99941, P18848, P51164, P18598, A8XAC6, Q03565, Q502P7, Q5FWL0, Q6P4Y1, P22035, E5AV36, E5AW43, E5AW45, Q9FLU1, Q8L4E7, P20293, Q6R3Q6, Q6RFL5, Q04787, Q3C1V8, Q810B3, P41726, P24648, P06545, P24649, P36627, Q13901, Q24572, P11821, P40121, P24452, P24029, P11819, P11820, P35203, A8AI78, A9KE65, A9NDK6, Q83CJ2, A7ZKA5, B7UNY3, B7MIE6, B7LFA9, B5YU43, Q7AFV7, B7NLC5, B7MPT2, B7M8Y3, B1X8V5, A7ZYV2, A1A9Q7, C4ZQC8, Q8FJ50, Q0TJ66, B1IV97, B7N3F5, B6I976, B1LJ04, Q1RDL6, B7LP19, Q1I490, B0KK26, A5W9N6, B1J5W7, Q87VN8, Q88DH7, B5F1Z5, A9MH53, Q57QP2, B5FR40, B5R049, B5R6G3, B4T2U5, B4TEN5, A9N6S2, Q5PGA2, C0Q893, B5BBH2, P63262, B4TSM3, P63263, A8GIL6, Q31YR1, Q32HR2, B2TTP8, Q0T634, Q7C254, Q3Z3C3, Q09184, Q2KJC1, Q99459, Q6A068, O08837, O02756, Q00322, Q03484, Q9N0J3, P49716, Q02637, Q9UFW8, Q8BHG9, Q3B7N1, Q7ZU90, Q86WJ1, A9X4T1, B6ZLK2, Q7KU24, Q9CXF7, O14646, O14647, E9PZM4, Q22516, O16102, Q12873, Q14839, Q6PDQ2, Q8TDI0, A2A8L1, D3ZD32, D3ZA12, Q06A37, A3KFM7, Q9P2D1, A2AJK6, B0R0I6, Q9HCK8, Q9JIX5, B5DE69, Q8BYH8, Q3L8U1, Q09XV5, O97159, P13656, A0A0H3MDW1, Q9HAW4, Q75BS7, B0Y8S0, Q2UUT4, A1CU75, Q6FQU2, Q0CSP9, Q4WLU1, Q2HHH2, P0CS57, Q1E6Q0, Q6BKH9, Q5B6U3, P0CS56, Q6CT00, Q59S45, A1DNV8, Q4PGT8, Q0UYV9, A7TL17, Q12510, A3LWH8, Q6C0U2, A5DAQ7, Q7S1H9, Q9FY74, Q6NPP4, Q9FYG2, O23463, Q8GSA7, Q9LSP8, A0RHK1, B7GGC4, C3L799, Q732P2, C1EP52, B7IUI7, B9IVB7, A7GRF8, C3P5N0, Q81WK7, B7HLG1, Q6HEY7, B7JJA6, Q819X8, Q9KA28, P39779, Q636J8, C0ZFA5, B7HDV1, A7Z4N7, Q5WFQ2, C3L0D4, A5I4L4, Q65JN2, A8FD81, Q8R9Y4, B2TJ39, B9E1I7, A6LSN9, A9VT66, B1II67, Q97I67, Q8XJQ5, Q0SSB9, Q895L3, A7FPZ9, A7GG24, C4L618, B1YIA8, B2V4F3, C5D8W0, A5N827, Q5L0N0, B1KWM6, C1FSK6, A4IM90, Q032T5, Q92C72, B8DG51, Q0TPQ2, Q8Y7J7, P59386, Q9CJ45, A2RHP2, A0AI83, Q2FHI3, Q2FZ27, Q2YXL3, C1L2I8, Q5HGH7, B9EBD5, Q720E1, P63844, Q6GHI0, A7X1N2, A6U173, A6QGF5, A5ISD9, P63843, P63845, A8Z3T5, Q5HPT7, Q4L5V7, Q6G9V8, P63846, B9DPG7, P63847, Q49X40, C0M7E5, B1HQV3, Q8CPG9, P0A349, Q04JG7, P59388, Q3JZL4, P0DA46, Q5XAC3, A8AVD7, C1C8H3, P0A351, B4U194, Q1J564, B1ID02, B8ZLU4, Q97PM1, A2RCV3, P0DA47, B2IRA0, B5XI03, Q8DP01, C0MEA0, A3CLF4, Q5LYE2, Q03J75, Q5M305, B9DVH6, C1CFG6, C1CSK2, B0K1T5, C1CLT1, B0K9V2, Q2KS10, P14269, P11115, P22058, O94166, O59958, Q4X0Z3, B8NGC8, A1C6L9, Q9HFS2, O94130, A2QCJ9, A1DH89, P27925, Q9P889, Q01981, Q05620, B0XSK6, P16220, O94131, C7GJZ2, E7KDM2, C8Z9U3, E7Q4T7, P38845, E7NIP0, B3LSR0, E7KPJ0, A6ZT54, E7LVH4, P9WMH3, G4RJY9, Q97Y88, Q57824, Q8NI51, A2APF3, P46963, P30999, P89105, Q13620, Q8LGH4, P15315, P41817, Q9P0U4, P27111, P22697, Q5A0W9, Q94AD1, P13483, P0A3H3, P17615, P0A3H6, Q9K7K5, P68573, P0A3H5, P02348, P68574, I1WEI8, P0ACF2, P0ACF1, P80605, P0A3H8, P0DMK4, P0A3H7, Q9HTL0, Q9LA96, P0A1R7, Q9KDA5, E0J6W8, P52680, Q9KV83, P0ACF6, P0A1R6, P28080, P0A3H4, P0ACF5, P0ACF3, P64388, P05384, P0A1R9, Q9KHS6, P0A1R8, P64389, Q9KQS9, Q9JR30, P52681, Q9ZD26, Q92HL4, P0ACF7, O67461, P0A3H2, O68451, Q44625, P0A3H1, Q57220, Q57153, Q45231, Q45722, Q45352, Q8KA69, Q89B22, Q57267, Q46121, P57144, Q5HUP6, P0CAV2, Q9Z8C7, B8GX11, Q9RZ89, P64386, P05385, P64387, P43722, Q9ZL08, O25506, Q9CI64, P29214, O33125, Q9ZHC5, P9WMK6, P9WMK7, Q9CK94, P02344, Q1RHD4, Q4UKH2, P05514, Q68XJ6, Q5HFV0, Q99U17, Q9XB18, Q7A5J1, Q6GGT8, Q6G990, Q7A0U9, Q92J57, Q9XB20, Q9XB22, Q9ZDZ2, Q9XB21, P0C0H3, P0DB64, P0DB65, P0C097, P96045, P73418, P02343, P02345, Q5XB35, P36206, P0C0H2, Q9PE38, Q87E48, O83278, Q9PQK9, Q10586, Q9M0V3, O49552, Q21554, Q805F9, P33194, B0M0P5, A1A4K3, Q9XYZ5, Q3U1J4, Q6QNU4, Q6NQ88, O13807, Q6P6Z0, Q2YDS1, Q5ZJL7, Q0VBY8, Q6E7D1, Q9ESW0, Q99J79, Q5R649, Q66JG1, Q59X49, P48732, P61990, Q96X56, O59632, Q5E7H1, P04497, P11806, P11807, Q65956, P36704, Q96687, P06500, Q8JN65, Q64759, P05662, O27652, P54103, P03198, Q3KSP1, Q1HVC1, P04293, Q85428, P24907, Q9YUS2, Q8YQL3, Q9R6T3, P0ABT2, P0C558, Q55024, P24274, P13320, Q8CCE9, P03191, P0C6Z0, Q3KSU3, P03203, Q3KST1, Q1HVG4, P38195, P34216, Q59X67, P13002, Q04688, P25032, Q09472, P18414, O14108, P29774, P29775, P29776, Q24312, P20222, Q9WV03, Q14320, Q05069, P97447, P64130, P64131, Q8Y231, Q8PD37, Q9HUW0, A6VM24, Q9PBY7, B6ENA5, A0KNY4, B8D9I3, P57480, Q8K9F0, Q87D75, A8AQG0, A4SJ84, A7MJA9, B7UJZ2, B8D7T5, A7ZSF6, P0A6R5, B7MC31, Q8PQ10, B5YSY6, B7LHW8, B7N0Q5, B7M0X3, C4ZSZ7, B7NLI7, A8A573, A1AGG1, B1XHN0, Q0TCJ5, P0A6R4, B1IQ31, B7NDN9, B1LGM6, Q1R667, B6I1Y1, A4WF77, B2VL73, C5BEX0, B7LRN6, Q4QLT0, A5UD91, Q7VNP1, A5UIB9, P44966, B8F6J8, B0UV86, Q0I1Y8, B5XND7, A6TES8, O52537, O54367, Q65V72, Q6DAJ8, P57902, C6DIJ6, B4EX21, Q6LLY3, P0CW85, O52540, A1SZI8, P0CW84, Q7N015, B5F7P5, A9MN98, B5FIW4, Q57J83, B5R1C8, B5REY3, B4SUP1, B4TJV9, Q5PJW3, B5BGT9, A9N889, B4TX93, C0PZT1, P0A6R6, P0A6R7, P0A6R9, A8GK78, B8E682, A3D9J7, A1SAM2, A6WTE7, A9L5E7, Q12S40, Q088K0, B0TJ39, A3Q9Q3, Q8EJR9, A8GZI0, A4YB21, B8CHY1, A8G0V0, A0KS72, Q0HN88, A1RFA1, B1KQE6, Q0HQJ9, B2U2N7, Q31W07, Q32B77, Q0T029, P0A6R8, Q3YWY8, Q2NWQ0, C4LAF3, A5F3S1, P64127, Q5E260, A7N080, P64128, B5FC67, P64129, Q7MGT8, B7VM54, A7FDQ0, A1JRL7, C3LQP6, B2K469, Q1C1P4, A9R1Z3, A4THB7, Q8ZAX8, Q1CDT5, B1JKF0, Q665E1, Q01167, O88621, P32314, P38196, P17678, P15976, P43429, P17679, P23771, P46153, P46152, Q92908, P18506, P24905, P27171, Q9WRL5, P19465, P39572, Q91661, Q91660, Q9Y692, Q9UKD1, P03825, P45756, P45758, P45759, P45757, P41441, P41442, P41443, P45760, P45761, P45762, P45763, P36678, P78347, Q8H181, Q9C882, P08286, Q02577, Q02575, Q02576, Q64221, P27709, P60008, D3ZZW6, Q9QYL0, Q05068, Q1RK52, Q9ZCL7, Q9AKA7, Q4UN06, Q92GN5, Q9AKF3, Q9AKK7, Q14527, Q9P0W2, Q9Z104, P48781, P19267, Q9SUP7, P26585, P40619, P26586, Q00423, Q10370, Q03973, P50483, P50484, O27731, Q03576, Q59041, Q9DE09, Q9NP08, O70218, P18818, Q5PCT5, P0ACG0, P09120, P18955, P0A1S3, P43841, P0A1S2, P57360, P0ACF9, P11065, Q45881, O14139, P38529, Q08DJ8, P38530, P38532, Q03933, P22335, P41152, P38531, P38533, D0VYS2, Q1HGE8, Q00613, Q9ULV5, Q9R0L1, P41153, Q5AQ33, P22121, Q8SS62, Q02953, P41154, C4V6H6, B7XIV9, Q40152, P10961, P22813, Q61286, P51514, Q99081, Q60420, Q28772, P51593, P46593, Q5AL03, Q9J5C9, Q6RZN2, O93117, P20498, Q77TL8, P32999, P16714, Q9QBG0, Q8V518, P08392, P41134, Q14602, P41135, Q9PWJ5, P20067, Q66J78, Q712G9, Q6PBD7, Q3ZC46, P41138, P41133, P41137, Q6GL62, Q4R5J7, P41136, P41139, Q2VIU1, Q5E981, Q02535, Q7ZXF3, P0A673, Q688C4, Q5RCH7, Q7SZ28, Q02363, P47928, Q91399, P9WMH1, Q9CCB5, Q06AV5, P9WMH0, Q9BTL4, B7SXM5, Q6P7D3, P17950, Q47GF4, Q47CN0, B7I698, B2HTI2, B0VV83, Q6F874, A3M2B0, B3H139, B0V5Q4, B0BNN9, A6VP80, A0KKP3, Q8UG61, B9JCZ0, A3MZX6, Q0VNG4, B7GZZ4, B6EN06, Q0AAM1, B9JV88, Q2IJA7, A7HBJ3, B4RS41, B8J836, B4UAP1, Q5P7Y1, A1K4E7, A1USJ0, Q6FZZ9, A8I5K8, Q6MMJ0, Q2KZM3, Q6G3K3, Q7WKR3, A9IVB2, Q7VVR6, Q7W7C5, A5EKG4, A4YW83, Q57DX3, Q2YNB8, B2S525, A9MAF7, C0RIB4, P64390, P64391, B0CLA3, B8D8T2, Q8KA11, B8D736, Q057Y8, P57231, B8H546, Q63TM8, B0T0T0, Q9A8I3, Q1QWK1, Q11J82, A8AHA5, Q7NYC0, B6IZG2, B6J7X5, A9KGB5, A9N8J9, Q47ZS6, A7MNY7, A9C3C8, P37982, A5EXT4, Q83C16, A8LLT5, B7US95, B7MAS3, A7ZMI0, P0A6X9, B7L6I5, B5YQ00, B7NT64, B7MVJ1, B7M1C1, C4ZYH4, B1XG19, A8A0Q4, Q0THB6, P0A6X8, B1IPL5, P0A6X7, B7N550, B6I8Q5, B1LE18, Q1RB83, C5B852, A4W9M9, B2VEL2, Q2N927, B7LQ77, Q7VLG4, Q4QKM2, A5UCA8, A5UF06, B8F4T7, P43723, Q0I3K4, Q2SDJ8, A1WU59, B0UU60, Q5QXL9, Q28RG2, B5XQD2, A6TAI1, Q5X1H9, A5IAL5, Q5ZS10, Q5WT88, Q2W4R6, P95516, Q65TL4, A1U2B7, Q0APH3, A6VYH5, Q60AY8, B7KYG6, B1ZKI7, A9W4E5, Q1GZS3, B1LZ99, Q9K4Q3, Q1D6D8, B0UR46, A9M383, B4RJZ4, Q5F9T5, P64393, Q0ADP0, Q9F297, Q1QMY9, P64392, Q3JBZ6, Q3SSS2, A1KSZ1, B6JGR8, A6X1X7, A1B366, Q2YBS0, Q6D4H4, C6DFZ2, B4RB18, Q9CN18, Q7N3Q2, Q82VV7, Q12BQ7, B4ETL2, A1VR71, Q15SX9, Q48JR7, Q02NN5, Q51472, A6V491, Q1IC09, Q3IIL3, B7V312, C3JZN1, Q4KEV8, Q3KEX6, A5W5D5, B0KKR2, A4XTS7, P0A127, B1J6V0, P0A126, Q4ZUG1, Q883H6, A4VM16, Q4FQ67, Q1Q8C9, Q8XZ23, Q2KA00, B3PVE1, A1SUQ7, B5ZXV9, Q1MIS5, Q982Z7, Q92QT3, C3M9J7, P30787, Q2IWQ7, Q21YS7, Q07MS0, Q6N676, Q214G0, Q136S3, B3QJM1, Q2RTS7, Q3J366, A4WTU0, Q1GI70, A3PJ63, Q21KD4, B5F7F6, Q5LQJ4, A9MFB7, Q57PU7, B5FJA2, B5RAW7, B5QVW2, B4T4N4, A9N236, B4TGH7, C0Q640, B5BA36, Q5PH86, B4TUF6, P0A1S1, P23302, P0A1S0, A1S700, B8E7F3, A8GDR1, A3D3R8, A6WMK6, Q12NT8, Q083K5, A9KYZ2, A3QF32, Q8EF98, B0TQY4, A8H5F1, A0KWC7, B8CR59, A4Y6H0, Q0HJ83, Q0HUQ0, A8FWI0, B1KRE5, Q321K4, B2U390, Q0T4S2, P0A6Y0, Q3Z260, A6U7Q6, Q32FI7, A1RK12, Q2NT26, A5VC87, Q1GT91, B4SQG8, B2FN73, Q3SK28, B8GRI6, Q31F20, C4LFG7, A5F1W7, C3LLR8, A7MVH4, B5FDX6, Q5E5G4, B7VPF6, Q87Q56, Q8DA35, Q9KSN4, Q3BRU3, P0A0U0, Q4UW51, P0A0T9, Q5GXY6, Q7MK44, B0RRH5, Q2P101, Q9PFD5, A7INL3, B2I9P2, B0U5D7, Q87AB7, A1JMM4, A7FHG7, Q1C735, B2K662, Q8ZDX2, A9R0A2, A4TIL7, Q9X9F6, B1JJ23, Q1CIH0, Q9RNZ5, A3N0A3, B0BP19, B3GXH5, A6VPK8, A0KJ94, Q8UIF9, A4SLS6, B9J885, P0A3I2, B9JZH2, B6EIY1, Q0AA51, A8IN83, C1DRR5, A9IL25, B2IKV6, Q89WE8, Q7W605, A5E8A7, B2S8E6, A1K4D5, Q57FM3, A9M798, A4YJI9, Q8YET3, A5VN89, C0RGK7, B0CIR1, B8D999, Q8G304, A9IJJ1, B8D7K1, Q2YP18, Q057N8, B1YV36, Q44654, Q1BY25, B1JXS2, A0K5M4, Q39IF8, P57394, A3MHP1, Q0BH90, A9ADV3, Q62M28, A1V6M0, A3NXW8, Q3JPY2, A2S4R7, A3NC29, B2JF07, B4EB39, Q63S07, Q2SY23, B2T634, Q13VC5, A4JCH7, B8H6A5, B0T273, Q9A2H5, Q11C35, Q1QVK5, Q7NTK9, A8AIH1, Q482G2, Q1LQG7, Q46Y54, Q47GJ8, P37983, A5EWQ2, A8LSF5, A7ZK01, B7UMZ8, B7MHM1, B7LDA4, P0A6Y3, B5YT46, B7NM62, B7MS27, C4ZQ38, B1X852, A7ZYL5, Q0TJE1, B7M841, P0A6Y2, B1IW19, P0A6Y1, B6I8Y4, B7NAR1, Q1RDU6, B1LJV1, C5BBR9, A4W8T1, B2VC76, B7LN74, A9H0B5, Q0BQI4, Q4QJU8, A5UBN4, Q7VLR8, A5UF83, P43724, B8F475, Q0I390, A1WUI6, Q2SCF7, B0UUH4, Q5QZ46, A6T1G0, Q28LB0, B5XY84, C1D546, A4G858, P95519, A6T704, A1TZF1, Q65SH8, Q0ATJ4, A6W0A0, Q608S8, P0A0U3, B8EMZ0, P0A0U1, P0A0U2, Q0AIZ2, Q82TD7, Q1QS30, A1KUD0, A6WV92, Q2Y7A9, Q3SWL5, B6JCN9, A1B8K9, A7HPD7, Q9CML8, Q6D404, C6DF68, Q6LPE4, B4ET28, Q48FN3, Q7N6D2, Q15T07, A6V2R4, Q02PW7, Q51473, B7VAL8, Q1ID97, C3K6K2, A4XTF3, Q3IL99, Q3K8V7, B0KTY3, A5W7F5, P0A128, P0A129, B1J5H2, Q885T0, Q4ZQ99, A1SYZ9, Q8Y0Y3, A4VMF7, Q1MM94, B3PZE1, Q2KD65, Q98GS0, B5ZN05, P80606, Q92SJ7, P0A3I1, Q06607, B6IUP4, Q2J2G1, Q07UH4, Q6NDN9, Q21CB6, B3Q602, Q13EQ5, Q9X4E2, A3PPV4, B9KU92, Q2RXP8, Q161H6, Q1GK81, Q21IT4, Q5LV98, B5F165, A9MHX0, Q57R19, B5FQ55, B5QZB6, B5R8J9, B4TD42, B4T146, A9N7V2, Q5PGG9, C0PXU8, B5BBP5, B4TRU3, P64395, P64394, P23303, A8GCH4, A1S6D6, B8EA98, A3D4A9, A6WNM7, A9L2X4, Q12ND8, Q081U7, A3QEC3, B0TT46, Q8EEI1, A8H4A7, A4Y729, A0KWP0, A8FVN3, Q0HIW8, Q0HV14, A1RJG1, Q31YT8, B2TUG9, B1KF50, Q0SX03, Q32E32, Q3Z3K9, A6U5F1, P0A6Y4, B4SSV3, Q2NUA6, C5BSK5, C4LF00, B8GRS4, A7MUP0, B2FNP6, A5F6Y4, Q5E3Z3, B5FG57, Q87N46, C3LNL8, B7VH32, Q8D8J3, Q7MLX5, Q8PK78, Q9KQT4, Q4UVD5, B0RSA5, Q8P8P6, Q2P3S1, B2SLH4, B2I6X0, Q9PAQ8, A1JMJ0, Q87BJ8, A7IHH0, Q1CA70, B2KA26, Q8ZGB1, A9R7I5, Q1CGG8, A4TN15, B1JRD6, Q66CI5, A7FJW6, O42410, O13089, Q13422, Q03267, Q9H2S9, Q9UKS7, Q5ZLR2, Q8BU00, Q9H5V7, Q6DBW0, A0JPB4, A4IFJ6, Q6NRM0, Q8C208, Q5R9W9, Q9UKT9, P24916, Q62655, Q9U6M2, P86937, Q9U6M1, P05412, Q9G051, P19188, P11340, P13468, Q8BN78, Q9TY84, Q9TY83, O61016, Q8IS98, Q07053, Q14807, Q8K339, P41727, P25960, Q6MZP7, Q571G4, Q641Z1, Q5RBN8, P21308, Q0VFI9, P50538, Q0VH34, Q05195, Q9BW11, Q7SX95, Q80US8, Q14582, Q60948, Q28DB3, Q0VH33, Q62912, O31644, Q66K74, P52162, P61245, P91664, P61244, P28574, P52164, P52161, Q07016, P0ACH8, Q9UH92, O08609, P27347, P13469, P50534, Q02395, Q9Y483, Q8U3J2, P50541, P50540, O09015, P50539, O13493, Q05935, P51960, P52550, P01105, P10243, P01104, P01103, P46200, P04197, Q08759, P10242, P06876, Q08856, P34061, Q6VNZ9, P13349, P17667, Q6SYV5, Q01795, Q91154, P15375, P24700, Q6PUV5, P23409, Q92020, Q7YS80, Q3YFL6, P24699, P19335, P34060, P17920, Q86VE0, Q8R4U1, Q08B72, Q01538, Q60430, Q13562, P06903, P06020, P46496, P09414, P14057, P19838, Q6P4R8, Q6P4L9, Q6PIJ4, Q12986, Q54S29, Q5E9S2, P18576, Q32KW0, P23708, P23511, P25207, Q6RG77, P63139, P25209, P63140, Q54WV0, P25208, P25211, P25210, Q5E9X1, P70353, Q5RA23, Q13952, Q62725, Q9UVL1, Q75B82, P0CO25, P11633, P11632, Q55C24, Q4WY33, Q6BRB4, Q6CC79, P0CO24, Q5B995, Q4PBZ9, Q4IQX3, P87057, Q6CVH3, Q7S045, P0A6Z6, P49011, Q9L9G1, O96028, Q12457, P0A4U6, P0A4U7, P78549, Q02818, Q02819, Q63083, P80303, Q9JI85, P81117, P52377, P28947, P84403, P10193, Q9E6Q7, P89432, Q6UDH3, Q9J3N7, P09299, P12959, Q89769, P0C9X7, P0C9X9, P0C9X8, P0C9Y0, Q65201, Q04073, Q77MR9, P89463, P10226, Q6UDI9, F5HID2, O52748, P24610, P23798, P35227, P46718, Q16342, P47816, Q2YDC9, Q08024, Q801X6, Q8ST83, A6ZM04, P03271, P9WI62, P9WI63, Q01842, O13852, P38193, P17742, P30044, Q9WTV0, P11825, P11826, P36385, P23724, P69529, P15285, Q9LZS0, Q9UJV8, Q8R4E6, P07256, Q8X487, Q96TL7, P42736, P10276, Q9ZWM9, P82280, Q00312, Q5ABZ2, Q5AB48, P03040, Q47588, Q05950, Q9P6H9, Q9XIB5, O80837, P32284, Q9BWE0, Q0VCC5, Q68H95, Q5U4E2, Q9X2V5, Q5A220, P48743, A6QLW9, Q5AMQ6, P48378, Q5EAP5, Q5RDR2, B2GV50, Q32NR3, B1WAV2, P48379, Q4R3Z4, Q33E94, P48382, Q9JL61, Q5RJA1, D2HNW6, Q8HWS3, Q2KHR2, Q8C7R7, Q9Z205, D3YU81, Q6ZV50, O14593, Q97ZZ8, O81242, Q10426, P0A7L3, P0CI78, Q5SHZ1, Q5SHQ0, Q02878, P38064, Q2FZ08, P25042, P13121, P13122, Q92766, P62844, P50889, P18392, Q08775, P42551, Q42337, P42553, P42552, Q7XLX6, P42554, Q93VI0, Q9NR83, P48383, Q2FVN3, Q01826, Q60611, Q9UPW6, Q8VI24, O14335, P11805, P24940, P23246, P24933, P11824, P11823, P06533, P52286, Q8TER0, Q5ZQU0, P25357, Q00910, P35710, O73790, P06843, Q6ZRS2, Q62093, Q05153, Q08943, P36631, P39015, P0ACG2, P0A1S4, P0ACG1, P0ACG3, P0A1S5, P08970, P31266, P46676, Q5ZLN5, Q28F51, P21675, Q9W7C2, P70326, Q5I2P1, Q3SA47, Q99593, Q9PWE8, Q04545, Q9PU53, P04445, Q01978, O27001, P56811, P14232, Q39237, Q39162, P14233, O24160, O43952, Q9FX53, Q39117, O80450, Q9LU92, Q9SDW0, Q96B42, P21580, Q60769, P13989, P15795, O14246, P10026, P07294, Q8VWK4, P05476, P14373, Q62158, P68351, P30674, P24911, Q9WRL6, Q9C7B1, Q9FFY9, P22265, P16525, P33122, Q00899, P25215, P28274, Q8UF86, O51777, Q9K6Y0, O66911, Q8YHC4, O34863, Q9PK60, O84337, Q9Z985, Q890X9, Q8XNI5, Q97LQ1, Q8G0I9, Q8X5U9, Q8FB02, Q46577, P0A698, P44410, Q7VLW2, Q9ZLD6, P0C0Z2, P56474, Q9CEL9, Q2YPX5, Q88YI7, Q72RM8, Q89L46, Q8F435, Q928A5, Q8Y4F6, Q71WU0, O26543, P63381, P13567, P47660, Q9CC24, P75176, Q8EUL1, P9WQK7, Q98PL2, P9WQK6, Q50968, Q9JUS4, Q9JZP1, P29927, P52087, P57979, Q8ENJ6, Q9HWG0, Q88QK7, Q98M36, P56899, Q1RK71, Q4UJW4, Q92G31, Q9ZCC3, Q68Y12, P0A196, P0A195, P0A699, Q5HHQ9, Q6GIN2, Q6GB71, Q8NXL9, P63383, P63382, Q8CPY9, Q5HQW9, P72481, Q829X3, Q99Y84, P0CZ40, Q9Z507, Q8NZJ2, Q5XA89, P63384, P0CZ41, P73412, P63385, Q9WYV0, O83527, Q9PR42, Q8DCJ3, Q87LA0, Q9KUW5, Q7MHB5, Q08518, Q56242, Q8PBH3, Q8PN26, Q8ZJ07, O31151, Q9PAR9, Q87BK9, P24912, P24913, P24915, P24914, P13342, Q12416, P9WF43, P28026, P0DKH5, E1B328, Q9SA80, Q9SV15, O22176, Q9LG05, Q9SVB7, Q9SZ67, Q9FL92, O04336, Q9FFS3, Q9SJA8, O04609, Q9SUS1, Q8VWJ2, Q9FL62, Q9FLX8, Q93WV0, O64747, O22900, Q8S8P5, Q8GWF1, Q93WT0, Q9XEC3, Q9C5T4, O22921, Q9S763, P59583, Q9SKD9, Q9ZUU0, Q9SR07, Q9ZSI7, Q8GY11, Q8VWQ5, Q9SHB5, Q8H0Y8, Q9SUP6, Q9FGZ4, Q93WU8, Q9SAH7, Q9C5T3, Q9C983, Q8VWQ4, Q8VWV6, Q9FHR7, Q9SK33, Q93WU9, O65590, Q93WY4, Q9CAR4, Q93WV5, Q9M8M6, Q9LZV6, Q9SJ09, Q93WV7, Q93WV6, Q9FYA2, Q9C557, Q32SG4, Q93WU6, Q9ZQ70, Q9LP56, Q9C6H5, Q9FG77, Q9SI37, Q9XI90, Q93WV4, Q9FL26, Q9STX0, Q9LY00, Q9C9F0, Q9LXG8, Q93WU7, Q9C519, O80462, Q9C516, C6KIE6, P03165, Q97CU3, Q9HM19, P41728, P41729, C3NGA4, O58787, Q05E29, Q8U1W7, B6YWX7, Q18DQ4, A6VIE7, A6URD8, Q12ZJ1, C5A636, Q5JGN3, Q971I0, Q4J8U0, A2STJ1, Q74NA9, A4G031, Q58958, A0B9G7, Q6M0W1, Q8TUY2, B1YAF2, C3MXG4, A0RYF8, A4WLS5, C4KII8, Q0W5G9, C3N7D0, C3MZB0, A7I9J0, C3MR75, A3CWZ8, Q9HNP3, Q6L2L3, O28211, O53509, Q2FTJ7, Q8ZTX7, Q5UXW7, Q980F8, B0R6V4, Q8TIN0, Q05067, Q05070, Q3INK4, A1RRN7, Q46FA5, A1RXF0, P9WKT7, P9WKT6, C6A1U6, B8GEU2, Q2NGR0, Q58103, A5UL35, A9A8D7, Q8PYQ7, Q54YS0, P56813, A6UVQ4, P12552, P0C5K9, P38197, P38194, Q9Y2T7, P21574, P25992, P53106, Q04013, P19268, C0H3Y4, P42547, P41730, P53107, P71036, C0H3S6, Q9H171, Q9QY24, Q8VDA5, Q92010, Q9Y2Y4, Q9JKD9, A1YGK1, Q5EXX3, A2T7E6, P15822, Q00900, A2A884, Q91689, Q3Y4E1, Q9UQR1, A0MS83, Q62806, Q61624, Q9Y2X9, Q8TD17, P32527 |
| **SSBs** | p81877, q9bww4, p09380, p09381, q9d032, q9cyz8, q9r050, p0age0, q04837, q9ry51, c0spb6, q9m9s3, p29558, p22336, q9bwg4, q00577, p27694, q98948, p03696, p69544, p9wgd5, q8vyf7, q9afi5, p03695, q9ll85, p54622, q13315, p68674, p03623, q3t0q6, p53996, o42395, p62634, p62633, q5r5r5, c1d1r8, q9rx92, q1j1n6, q9ry80, p03264, o36360, q03444, p03227, q1hvb8, q3ksn9, q66611, p03265, p36384, p52338, p24910, p52538, p30672, q18lf9, q9wrl7, p52339, p13215, p04415, p04995, q9usu3, p69542, p03670, o80294, p03672, p69543, p15417, p68676, p03671, p68672, p68670, q9w3m9, q04832, q14103, p61980, p41453, q65365, q9uxg1, q4r8g6, q8n635, b0bmx9, q9d513, p34496, q8c854, q14249, q96yr4, q8gxh3, q8gwj4, q9sx99, q9fyj2, q96rr1, p07271, p03692, p78527, p42669, p86252, q9skz1, q06609, p0aaz4, q3zbp3, q91w59, q5pqp1, q9rt63, q38617, q23696, q24492, q01588, q92372, p15927, q92373, q92374, o14087, p32445, p09651, q58559, o27438, b7fas6, q6nrf9, q6de02, a6qlk2, q9bq15, q8r2y9, q66k94, q3swt1, q5prc7, a5d7p8, q96ah0, q8bgw5, q5fvp2, q5zj32, q2nkt2, q7zv26, q9nry2, q8avv6, b5kfm4, b9eq30, q3txt3, q9cgs5, q92fr5, q8yar8, q8kb47, q97kh4, p0a4k0, p0a2f7, p0a2f6, q5his8, q931k4, q847g1, q6g7v6, p66850, p66851, q5xe77, q8p2h3, q82fg5, q55499, q83n34, q9kyi9, q83nu2, q9phe7, q8kam2, p28043, q9cdm9, q8y4x1, q92fk7, q8vmm4, q9rhf4, q97ht8, q8zsd2, q93gp7, q5hj26, q6gca6, q8e7h6, q932a8, q8e220, q82ci4, q9x8u3, q5xa62, p73145, p66857, q8nzj0, p66856, q97cx3, q9pdi7, q928x8, p28044, q8dxi7, p37455, p18310, q84j78, q32pb0, p18022, q9cyr0, q95kk4, p28045, p28042, p0a4k1, q8gan5, q8uf87, q814g6, q8ksb6, q8e0z9, q9k5n9, q81ji3, q8a7m7, p66847, q5rdq0, p59928, q8g757, p66846, q9xjg4, q37885, q38504, q89l50, p06953, q2ypx7, q8yhc2, o66475, q8g0j1, p0c118, q89a53, p57610, o51141, q8r6m2, o69302, q8k933, q823k0, q9a894, q9pkz4, o84048, q9z8f7, q8xh44, q899r2, q8nlg0, q8flp9, q83ep4, p59927, p0age2, p0age1, q839y9, p59930, q8re26, p44409, q9zjy2, p59931, o25841, q890k1, q72uu3, q8f052, p0a611, p47337, p46390, q8ewt6, q98pv9, p75542, p9wgd4, p66848, p66849, q82s98, q8cx55, p60471, q9cjp4, p28046, q8l2a6, p40947, q88qk5, q889u1, q8y2b4, q98m41, p56898, q9zaq8, q1rk72, q92g30, q9zcc2, q4ujw3, p59932, q68y11, p25762, p0age3, p77953, q6gf73, q8ea81, q8nvn2, q8cnk0, q99sq9, q8dsd8, p66852, p0df76, p66854, p0df77, p66855, q97w73, q9kh06, q8diu1, q5slp9, q9wz73, o85824, o83101, q9ppt7, q9kuw2, q8d254, q87la3, q8dcj0, q8pij2, p59933, q8p778, q87dq5, q8zj06, p11031, p28009, p25735, p20703, p03698, o21949, o21947, o21952, o21953, o21948, o21954, o21950, o21956, o21959, p09035, o21945, p20313, o21955, o21957, o21960, p68675, o21958, o21951, p09797, o21946, p08062, p0a3w8, p17637, b2lxs7, q66gr6, d9j034, p51237, p49536, q1xdq0 |

,
