# Supplementary material for: Analysis and prediction of single-stranded and double-stranded DNA binding proteins based on protein sequences
Source: BMC Bioinformatics. 2017 Jun 12;18:300. doi: 10.1186/s12859-017-1715-8 (PMC5469069; doi:10.1186/s12859-017-1715-8)
Supplement: Supplementary file 2 — This file contains the list of UniProt codes for non-redundant DNA-binding protein sets from UniProtKB/Swiss-Prot (www.uniprot.org). (DOCX 19 kb) [file 12859_2017_1715_MOESM2_ESM.docx]

**Table S2 -** **the list of UniProt codes for non-redundant DNA-binding protein sets.**

| **Types** | **ID** |
| --- | --- |
| **DSBs** | a0a0h3, a0b9g7, a0ms83, a0rhk1, a0ryf8, a1b366, a1cu75, a1l4x7, a1rxf0, a1suq7, a1syz9, a1szi8, a1vr71, a1wui6, a2a884, a2apf3, a2stj1, a3cwz8, a3lwh8, a5daq7, a5ewq2, a5ext4, a5ul35, a5vc87, a6uvq4, a6vie7, a6vm24, a6vp80, a6vpk8, a6vyh5, a6w0a0, a6zm04, a7hpd7, a7i9j0, a7tl17, a7zkt2, a8gil6, a8xac6, a9ke65, a9m383, a9x4t1, b0m0p5, b0r0i6, b1yaf2, b2i6x0, b5de69, b6ena5, b6izg2, b6zlk2, b7i698, b7kyg6, b7sxm5, b7xiv9, b8d8t2, b8d9i3, b8e682, b8h546, c0h3s6, c0h3y4, c3l0d4, c3nga4, c4lfg7, c4v6h6, c6kie6, c7gjz2, d0vys2, d2hnw6, d3yu81, d3zzw6, e1b328, e5av36, e5aw43, e5aw45, f5hid2, g4rjy9, i1wei8, o04336, o04609, o13046, o13089, o13493, o13807, o13852, o14108, o14139, o14246, o14335, o14593, o14647, o16102, o22130, o22176, o22812, o22900, o22921, o23463, o23620, o24160, o26543, o27001, o27652, o28211, o31151, o31644, o43918, o43952, o51777, o52748, o53509, o58787, o59958, o61016, o64747, o65590, o66911, o67461, o73790, o75717, o80450, o80462, o80837, o81242, o83278, o83527, o88621, o94130, o94131, o94166, o96028, o97159, p01103, p01105, p02343, p02345, p03040, p03084, p03085, p03165, p03191, p03198, p03271, p03825, p04197, p04293, p04445, p04497, p04498, p05385, p05412, p05476, p05662, p06020, p06022, p06533, p06843, p06903, p07256, p08286, p08392, p08821, p08874, p08970, p09414, p0a0u3, p0a128, p0a3h3, p0a3h6, p0a3h8, p0a3i2, p0a4u6, p0a673, p0a6z6, p0a7l3, p0abt2, p0acf0, p0acf4, p0acf8, p0acg2, p0ach8, p0c558, p0c5k9, p0cav2, p0ci78, p0co25, p0cs57, p10026, p10226, p10242, p10276, p10961, p11065, p11115, p11340, p11633, p11805, p11807, p11820, p11821, p11824, p11826, p11938, p12552, p12959, p13002, p13121, p13123, p13320, p13342, p13468, p13469, p13483, p13567, p13656, p13989, p14232, p14233, p14373, p15285, p15315, p15795, p15822, p15976, p16220, p16525, p16951, p17615, p17678, p17742, p17920, p18392, p18414, p18506, p18598, p18848, p19188, p19268, p19436, p19465, p19838, p20067, p20222, p20293, p21308, p21538, p21574, p21580, p21675, p22035, p22058, p22121, p22265, p22335, p22697, p22813, p23246, p23511, p23724, p23771, p24029, p24274, p24452, p24610, p24649, p24905, p24907, p24911, p24912, p24913, p24914, p24915, p24916, p24933, p24940, p25032, p25042, p25209, p25210, p25215, p25357, p25960, p25992, p26585, p26586, p27111, p27171, p27347, p27709, p28026, p28274, p28947, p29214, p29383, p29774, p29775, p29776, p30044, p30674, p30999, p31266, p31441, p32284, p32314, p32447, p32504, p32527, p33122, p33224, p34216, p34233, p35203, p35227, p35251, p35710, p36206, p36385, p36627, p36631, p36678, p36704, p38064, p38065, p38193, p38194, p38195, p38196, p38197, p38529, p38530, p38531, p39015, p39572, p40619, p40969, p41139, p41152, p41153, p41154, p41441, p41442, p41443, p41726, p41727, p41728, p41729, p41730, p41817, p42547, p42551, p42736, p43841, p44410, p45756, p45757, p45758, p45759, p45760, p45761, p45762, p45763, p46152, p46496, p46593, p46676, p46963, p47660, p48378, p48382, p48383, p48732, p48743, p48781, p49011, p49716, p50534, p50538, p50539, p50541, p50889, p51514, p51593, p52087, p52161, p52286, p52377, p52550, p53106, p53107, p54103, p57144, p57360, p59328, p59583, p60008, p60848, p61244, p61798, p61799, p61800, p61802, p62844, p64130, p64388, p68351, p68573, p68767, p69529, p71036, p73412, p73418, p78347, p78549, p80303, p82280, p86937, p87057, p89105, p89432, p91664, p95516, p97447, p9wf43, p9wi62, p9wkt7, p9wmh3, p9wmk6, q00423, q00613, q00899, q00900, q00910, q01167, q01538, q01795, q01826, q01842, q01978, q02395, q02486, q02575, q02577, q02637, q02818, q02878, q02953, q03267, q032t5, q03576, q03933, q03973, q04013, q04073, q04545, q04688, q04787, q04jg7, q05067, q05068, q05069, q05070, q05153, q057n8, q057y8, q05935, q05950, q05e29, q06481, q06a37, q07016, q07053, q08024, q08702, q08759, q08775, q08856, q08943, q08b72, q09184, q09472, q09xv5, q0aiz2, q0aph3, q0atj4, q0bqi4, q0uyv9, q0w5g9, q10426, q10586, q12416, q12457, q12873, q12986, q12zj1, q13422, q13562, q13620, q13901, q13952, q14527, q14582, q14807, q14839, q16342, q16531, q18dq4, q1e6q0, q1gt91, q1hge8, q1hvg4, q1i490, q1qvk5, q1rhd4, q1rk52, q21554, q22516, q24312, q24572, q28db3, q2fhi3, q2ftj7, q2fvn3, q2fz08, q2hhh2, q2iwq7, q2khr2, q2kjc1, q2ks10, q2kzm3, q2n927, q2nb98, q2ngr0, q2sdj8, q2w4r6, q2yds1, q31f20, q32sg4, q33e94, q39117, q39237, q3ink4, q3l8u1, q3swy1, q3u108, q3y4e1, q44625, q44654, q45881, q46121, q46577, q47588, q47gf4, q4fq67, q4pbz9, q4pgt8, q4qkm2, q4ujw4, q4v3e0, q502p7, q50968, q54s29, q54wv0, q54ys0, q55c24, q56242, q57824, q58103, q58958, q59041, q59s45, q59x49, q59x67, q5a0w9, q5a220, q5ab48, q5abz2, q5al03, q5amq6, q5aq33, q5b995, q5e7h1, q5eap5, q5exx3, q5lye2, q5rja1, q5shq0, q5shz1, q5zjl7, q5zln5, q5zs10, q60ay8, q61286, q62093, q62431, q62655, q62912, q63003, q63tm8, q64759, q65201, q65956, q66j78, q66jg1, q66k74, q688c4, q6bkh9, q6c0u2, q6dbq1, q6dbw0, q6e7d1, q6g3k3, q6gl62, q6l2l3, q6mmj0, q6mzp7, q6npp4, q6nq88, q6nrm0, q6p0e7, q6p4l9, q6p4r8, q6p4y1, q6rfl5, q6rzn2, q6udh3, q6udi9, q6vnz9, q6vuc0, q6zrs2, q6zv50, q712g9, q72rm8, q74na9, q75bs7, q77mr9, q7ku24, q7sx95, q7t287, q7w605, q7xlx6, q7yrz2, q7z2e3, q7zu90, q801x6, q810b3, q82vv7, q85428, q86ve0, q86wj1, q88yi7, q890x9, q89769, q89b22, q89l46, q8bm75, q8bn78, q8bu00, q8c208, q8cce9, q8enj6, q8eul1, q8gsa7, q8gwf1, q8gwq2, q8gxb3, q8gy11, q8h0y8, q8h181, q8is98, q8jn65, q8k339, q8k9f0, q8l4e7, q8l7l5, q8lgh4, q8msg8, q8ni51, q8pbh3, q8pd37, q8r4e6, q8r9y4, q8s8p5, q8ss62, q8st83, q8td17, q8td26, q8tdi0, q8ter0, q8tin0, q8tuy2, q8u3j2, q8ug61, q8vwj2, q8vwk4, q8vwq4, q8vwq5, q8vwv6, q8vyj2, q8x487, q8y0y3, q8y231, q8yql3, q91154, q91660, q91661, q91689, q91zk0, q92010, q92466, q92481, q92754, q92766, q92908, q92sj7, q93wt0, q93wu7, q93wu8, q93wu9, q93wv0, q93wv4, q93wv5, q93wv6, q93wv7, q93wy4, q940i0, q94ad1, q96b42, q96tl7, q971i0, q97cu3, q97y88, q97zz8, q98pl2, q99941, q99j79, q9btl4, q9bw11, q9bwe0, q9c516, q9c519, q9c5t3, q9c5t4, q9c6h5, q9c7b1, q9c882, q9c983, q9c9f0, q9car4, q9ci64, q9cxf7, q9de09, q9e6q7, q9ffy9, q9fg77, q9fgz4, q9fhm5, q9fhr7, q9fir1, q9fl26, q9fl62, q9fl92, q9flu1, q9flx8, q9fx53, q9fy74, q9fya2, q9fyg2, q9g051, q9h171, q9haw4, q9hfs2, q9hnp3, q9htl0, q9huw0, q9j3n7, q9j5c9, q9jkd9, q9jl61, q9jr30, q9k4q3, q9k7k5, q9l9g1, q9lg05, q9ljg8, q9lp56, q9lsp8, q9lta2, q9lvb0, q9lxg8, q9ly00, q9lzs0, q9lzv6, q9lzx7, q9m2s3, q9m8m6, q9n0n3, q9np08, q9nr83, q9p0u4, q9p0w2, q9p2d1, q9p6h9, q9pe38, q9pk60, q9pqk9, q9pr42, q9pu53, q9pwe8, q9qy24, q9qyl0, q9r6t3, q9rnz5, q9rz89, q9s763, q9s7c9, q9sa80, q9sah7, q9sb31, q9sdw0, q9shb5, q9si37, q9sj09, q9sjg4, q9sk33, q9skd9, q9sr07, q9sr17, q9stx0, q9sup6, q9sup7, q9sus1, q9sv15, q9svb7, q9syg2, q9sz67, q9sz70, q9ty84, q9u6m1, q9u6m2, q9ufw8, q9uh92, q9ukd1, q9uks7, q9ukt9, q9ulv5, q9upw6, q9uvl1, q9wrl5, q9wrl6, q9wtv0, q9wv03, q9wyv0, q9x2v5, q9xi90, q9xib5, q9xyz5, q9y2t7, q9y2x9, q9y2y4, q9y483, q9y692, q9yus2, q9z0e3, q9z205, q9z507, q9z8c7, q9z985, q9zcl7, q9zd26, q9zl08, q9zld6, q9zq70, q9zsi7, q9zuu0, q9zwm9 |
| **SSBs** | q9bww4, q9ry51, c0spb6, p29558, p22336, q00577, p27694, q98948, p03696, p69544, q8vyf7, p03695, q9ll85, p54622, q13315, p68674, p03623, p53996, q9rx92, q9ry80, p03264, o36360, q03444, p03227, q66611, p36384, p52338, p24910, p30672, q18lf9, q9wrl7, p52339, p13215, p04415, p04995, q9usu3, p03670, o80294, p03672, p15417, p68676, p03671, p68672, p68670, q9w3m9, q04832, q14103, p61980, p41453, q65365, q9uxg1, q4r8g6, p34496, q8c854, q14249, q96yr4, q8gxh3, q8gwj4, q9sx99, q9fyj2, q96rr1, p07271, p03692, p78527, p86252, q9skz1, q06609, p0aaz4, q9rt63, q38617, q23696, q24492, q92372, p15927, q92373, q92374, o14087, p32445, p09651, q58559, o27438, b7fas6, q8r2y9, q5prc7, a5d7p8, q8avv6, q92fr5, q8kb47, q97kh4, p0a4k0, q5his8, q5xe77, q82fg5, q55499, q83n34, q9kyi9, q9phe7, q8kam2, p28043, q9cdm9, q92fk7, q8zsd2, q93gp7, q932a8, q82ci4, p73145, p66857, q97cx3, q8dxi7, q84j78, q9cyr0, q8ksb6, q81ji3, q8a7m7, p66847, q8g757, q9xjg4, q37885, q38504, o66475, q89a53, p57610, o51141, q8r6m2, o69302, q823k0, q9a894, o84048, q8xh44, q899r2, q8flp9, q83ep4, p59927, q839y9, p59930, q8re26, q9zjy2, p59931, q890k1, q72uu3, p47337, p46390, q8ewt6, q98pv9, p75542, p66848, q82s98, q8cx55, p60471, q8l2a6, p40947, q889u1, q8y2b4, q98m41, p56898, q9zaq8, q92g30, p59932, p77953, q8ea81, q8cnk0, q97w73, q8diu1, q9wz73, o85824, o83101, q9ppt7, q8d254, q8dcj0, q8pij2, p59933, p11031, p28009, p20703, p03698, p08062, p17637, b2lxs7, q66gr6, d9j034, p51237, p49536, q1xdq0 |

,
