# Supplementary material for: Analysis and prediction of single-stranded and double-stranded DNA binding proteins based on protein sequences
Source: BMC Bioinformatics. 2017 Jun 12;18:300. doi: 10.1186/s12859-017-1715-8 (PMC5469069; doi:10.1186/s12859-017-1715-8)
Supplement: Supplementary file 3 — This file contains the list of PDB codes for non-redundant DNA-binding protein independent sets from PDB (www.rcsb.org/pdb/). (DOCX 16 kb) [file 12859_2017_1715_MOESM3_ESM.docx]

**Table S3 -** **the list of PDB codes for non-redundant DNA-binding protein independent sets.**

| **Types** | **ID** |
| --- | --- |
| **DSBs** | 1aa3a, 1b00a, 1b8za, 1bm9a, 1bw6a, 1bxia, 1d8ja, 1e7da, 1fx7b, 1h0xb, 1hw1b, 1jg6a, 1jihb, 1jt6e, 1jy1a, 1ku2b, 1okrb, 1q0sa, 1rwza, 1smnb, 1tfra, 1vdeb, 1wtdb, 1xhxb, 1ynma, 1ysea, 1z5zb, 1zaeb, 1zlje, 1zpqb, 2ay0e, 2ckxa, 2eb7a, 2h8ea, 2hzab, 2iuse, 2oa9b, 2odha, 2p5ka, 2polb, 2qsfa, 2qsha, 2qsha, 2r0qe, 2r5yb, 2r8jb, 2r9lb, 2rbab, 2rbfb, 2rs6a, 2v1ua, 2v6eb, 2ve9e, 2vlaa, 2w36b, 2w42b, 2w7nb, 2wj0b, 2wwyb, 2yvhb, 2z3xb, 2zo2b, 3a46b, 3a5tb, 3aafb, 3bamb, 3bepb, 3biea, 3bm3b, 3brfd, 3brgc, 3bs1a, 3btxa, 3buee, 3c25b, 3c2ia, 3clcb, 3cmxd, 3cmya, 3crol, 3crxb, 3cvva, 3cwsb, 3dfxb, 3dlra, 3dw9b, 3e54b, 3e6cc, 3eh8d, 3ei1b, 3eikb, 3eyib, 3f23b, 3f27d, 3f2ba, 3fc3b, 3fdqb, 3fhze, 3gfic, 3glie, 3gnba, 3gp1a, 3gv8b, 3gvab, 3gxqb, 3gz5b, 3gz6b, 3h0db, 3hosb, 3hqfa, 3hx0k, 3i0wa, 3igmb, 3jxbd, 3k0sb, 3k4xa, 3k5nb, 3k9fb, 3kdec, 3kmdc, 3kmpb, 3kntb, 3l2ub, 3orca, 3v68a |
| **SSBs** | 1a1va, 1ak0a, 1bj6a, 1h95a, 1i7da, 1i8mb, 1j4wa, 1jb7b, 1jmsa, 1kxla, 1mw9x, 1p4db, 1qzgb, 1xjva, 1yuaa, 2a0ia, 2b3gb, 2bpa2, 2cczb, 2hqle, 2pi2e, 2py5b, 2up1a, 2vtbe, 2vw9b, 2wkda, 3a5ub, 3c95a, 3g2cb, 3khcb, 3pgzb, 3q8da, 3qk2a, 3sxub, 3tekb, 3u50c, 3u58b, 3u7fb, 3ubyb, 3ulpb, 4a75e |

,
