# Supplementary material for: Analysis and prediction of single-stranded and double-stranded DNA binding proteins based on protein sequences
Source: BMC Bioinformatics. 2017 Jun 12;18:300. doi: 10.1186/s12859-017-1715-8 (PMC5469069; doi:10.1186/s12859-017-1715-8)
Supplement: Supplementary file 4 — This file contains the results of leave-one-out cross validation. (DOCX 24 kb) [file 12859_2017_1715_MOESM4_ESM.docx]

**Table S1** The performance of different kinds of feature descriptors in non-redundant dataset using leave-one-out cross validation procedure by random forest method.

| Features | ACC | SN | SP | AUC | MCC | F1 |
| --- | --- | --- | --- | --- | --- | --- |
| OAAC | 0.857 | 0.901 | 0.822 | 0.940 | 0.718 | 0.857 |
| Dipeptide=0 | 0.833 | 0.908 | 0.795 | 0.902 | 0.705 | 0.819 |
| Dipeptide=1 | 0.848 | 0.924 | 0.795 | 0.911 | 0.708 | 0.847 |
| Dipeptide=2 | 0.842 | 0.906 | 0.796 | 0.902 | 0.693 | 0.872 |
| AAindex | 0.839 | 0.908 | 0.790 | 0.906 | 0.688 | 0.838 |
| PSSM | 0.850 | 0.904 | 0.808 | 0.916 | 0.706 | 0.849 |
| All features | 0.860 | 0.927 | 0.811 | 0.920 | 0.729 | 0.859 |

**Table S2** The performance of different kinds of feature descriptors in non-redundant dataset using leave-one-out cross validation procedure by SVM method.

| Features | ACC | SN | SP | AUC | MCC | F1 |
| --- | --- | --- | --- | --- | --- | --- |
| OAAC | 0.760 | 0.907 | 0.690 | 0.778 | 0.557 | 0.780 |
| Dipeptide=0 | 0.683 | 0.970 | 0.614 | 0.683 | 0.462 | 0.650 |
| Dipeptide=1 | 0.609 | 0.902 | 0.626 | 0.649 | 0.403 | 0.541 |
| Dipeptide=2 | 0.714 | 0.980 | 0.638 | 0.714 | 0.514 | 0.690 |
| AAindex | 0.746 | 0.980 | 0.663 | 0.746 | 0.571 | 0.729 |
| PSSM | 0.773 | 0.828 | 0.732 | 0.783 | 0.602 | 0.762 |
| All features | 0.780 | 0.852 | 0.733 | 0.780 | 0.572 | 0.778 |
